# Toxicity of PEG-Coated CoFe_2_O_4_ Nanoparticles with Treatment Effect of Curcumin

**DOI:** 10.1186/s11671-018-2468-7

**Published:** 2018-02-14

**Authors:** Shahnaz Akhtar, Wenzhen An, Xiaoying Niu, Kang Li, Shahzad Anwar, Khan Maaz, Muhammad Maqbool, Lan Gao

**Affiliations:** 1 School of Life Sciences, Lanzhou University, Lanzhou, Gansu 730000 People’s Republic of China; 2 Key Laboratory of Nonferrous Metals Chemistry and Resources Utilization of Gansu Province, Lanzhou University, Lanzhou, Gansu 730000 China; 3 Department of Physics, Islamia College Peshawar (Chartered University), Peshawar, 25120 Pakistan; 4 Nanomaterials Research Group, Physics Division, PINSTECH, Nilore, Islamabad, 45650 Pakistan; 5 Department of Clinical and Diagnostic Sciences, The University of Alabama, Birmingham, AL 35294-1212 USA

**Keywords:** Nanoparticles, Hydrothermal technique, Annealing, Toxicity, PEG-coated CoFe_2_O_4_

## Abstract

In this work, CoFe_2_O_4_ nanoparticles coated with polyethylene glycol (PEG) were successfully synthesized via a hydrothermal technique. Morphological studies of the samples confirmed the formation of polycrystalline pure-phase PEG-CoFe_2_O_4_ nanoparticles with sizes of about 24 nm. Toxicity induced by CoFe_2_O_4_ nanoparticles was investigated, and biological assays were performed to check the toxicity effects of CoFe_2_O_4_ nanoparticles. Moreover, the healing effect of toxicity induced in living organisms was studied using curcumin and it was found that biochemical indexes detoxified and improved to reach its normal level after curcumin administration. Thus, PEG-coated CoFe_2_O_4_ synthesized through a hydrothermal method can be utilized in biomedical applications and curcumin, which is a natural chemical with no side effects, can be used for the treatment of toxicity induced by the nanoparticles in living organisms.

## Background

The use of nanoparticles (NPs) offers many advantages due to their unique chemical and physical properties which are substantially different from their bulk counterparts [1]. Cobalt ferrite (CoFe_2_O_4_) as one of the most important magnetic materials has aroused immense interest at the nanoscale because of its various applications in recent technologies [2–5]. It is considered as one of the competitive candidates for its wide range of applications, mostly in medical industry, due to its capability to possess the desired physical and chemical properties at the nanoscale. Moreover, CoFe_2_O_4_ is easy and cost-effective to be fabricated with controlled composition, shape, and size required for a particular application. In this regard, the diameter of CoFe_2_O_4_ nanoparticles for biological applications below 100 nm can greatly influence the physiochemical properties and pharmacokinetics in living organisms. Larger particles with a diameter greater than 100 nm are used as a contrast agent for magnetic resonance imaging of the gastrointestinal tract, while smaller particles, below ~ 20 nm, are used as carriers for tumor treatments. For clinical application of cobalt ferrite nanoparticles, it is very important to investigate the biosafety both in vivo and in vitro [6, 7]. Many nanoparticles taken via oral or intravenously into the body are mainly distributed in the liver, kidney, and lung so as to lead to various inflammations in these organs. Compared to other materials, cobalt ferrite has not been studied extensively to explore its toxicity in living organisms and then its healing effect using curcumin, although few other works have been reported on investigating the toxicity and biosafety of polyethylene glycol (PEG)-coated cobalt ferrite nanoparticles.

From the toxicity point of view, the main concern is the excessive exposure that requires elimination of accumulated nanoparticles from biological organs as well as it requires urgent treatment of inflammatory disorders. Some researchers have tried to study several anti-inflammatory drugs on the treatment of toxicity of nanoparticles in vivo, and they found that these anti-inflammatory drugs could promote the excretion of nanoparticles that are accumulated in the body to a certain extent in order to reduce or eliminate the tissue inflammatory effects [8, 9]. *Curcuma longa* (turmeric) is a traditional medicinal herb with a quite long history of its use as a treatment for inflammatory diseases in Southeast Asia. Numerous studies have been reported on the antioxidant properties, antimutation and antitumor effects, and carcinogenic characteristics of curcumin [10, 11]. Curcumin has the ability of healing wounds as well as treating the liver ailments, urinary tract diseases, and hepatitis [11]. It alleviates oxidative stress and inflammation in chronic diseases through the Nrf2-keap1 pathway. Curcumin can suppress pro-inflammatory pathways related with most chronic diseases and blocks both the production of TNF and the cell signaling mediated by TNF in various types of cells. Moreover, curcumin may also act as a TNF blocker from in vitro and in vivo by binding to TNF directly [12].

In this study, we have successfully prepared PEG-coated CoFe_2_O_4_ nanoparticles with a controlled shape and size of about 25 nm using a hydrothermal technique. After giving different exposures (doses) of CoFe_2_O_4_ nanoparticles, we have examined blood analysis, HE staining, and biodistribution as well as the treatment effect of curcumin on the toxicity caused by PEG-CoFe_2_O_4_ nanoparticles. This study presents a new approach to investigate the toxicity effect of CoFe_2_O_4_ nanoparticles and then the treatment of the toxicity caused by PEG-CoFe_2_O_4_ nanoparticles in vivo using curcumin.

## Methods

### Preparation of Cobalt Ferrite Nanoparticles

Cobalt ferrite nanoparticles were synthesized using a hydrothermal technique. For this purpose, an adequate amount of ferric nitrate and cobalt chloride was dissolved in deionized water and then mixed with aqueous solutions of PEG and sodium hydroxide (NaOH). Double-distilled deionized water was used as the solvent to avoid the presence of any impurities in the final nanoparticles. The mixture was stirred for about 30 min using a magnetic stirrer and then poured into the autoclave and heated for 6 h at 180 °C to perform the hydrothermal reaction. After the reaction was completed, the product was cooled to room temperature and then washed twice with deionized water and then with ethanol to remove the excess PEG and other un-dissolved salts, if present in the solution. Finally, the product was dried at 80 °C overnight and then ground into powder to get the desired cobalt ferrite nanoparticles. In this stage, the nanoparticles were found amorphous which was confirmed by the XRD shown in Fig. 2a. To get the nanoparticles in crystalline form, the samples were then annealed at 500 °C for 6 h and the final product was obtained in the form of crystalline PEG-CoFe_2_O_4_ nanoparticles that was confirmed by the XRD shown in Fig. 2b.

### 99mTc Labeling of PEG-CoFe_2_O_4_ Nanoparticles

Radiolabeling of PEG-coated CoFe_2_O_4_ nanoparticles was performed with 99mTc using stannous chloride (SnCl_2_) as the reducing agent and dissolved the nanoparticles in deionized water under ultra-sonication conditions for about 0.5 h. SnCl_2_, ascorbic acid, and 99mTcO_4_ were then added into the nanoparticle suspension (with cobalt ferrite of ~ 0.4% by weight). For accurate data, the radioactive counts were measured within 24 h due to the short lifetime of 99mTc (~ 6 h). The pH of the mixture was adjusted in the range 5–10 using 1.0 M NaHCO_3_ solution; then, suspension of PEG-CoFe_2_O_4_ was added to it and the resultant mixture was then stirred at 10,000 for 25 min at 80 °C. After centrifugation, the supernatant was decanted, and the remaining material was identified to be 99mTc PEG-CoFe_2_O_4_. Paper chromatogram (under the chromatographic solutions of normal saline and acetone) was used to measure the yields of the labeled compounds. The radioactive labeling yield of the nanoparticles was found to be around 70% that reflects the real distribution and metabolism in vivo.

### Biodistribution of PEG-CoFe_2_O_4_ Nanoparticles

Kunming mice weighing in the range 15–18 g were provided by the Laboratory Centre for Medical Science, Lanzhou University, Gansu, People’s Republic of China. All animals were housed in individual cages with a temperature-controlled system (21 to 22 °C), and lights were switched on from 08:00 to 20:00 hours. Proper food and water were given to the mice as recommended according to the animal protocols by the European Communities Council Directive of November 24, 1986 (86/609/EEC), and approved by Institutional Animal Care and Use Committees of Gansu Province Medical Animal Center and Lanzhou University Animal Committees Guideline (China). The mice were divided randomly into seven groups (five mice/group), injected intravenously with 99mTc-PEG-CoFe_2_O_4_ solution, and then killed at 1, 6, 16, and 24 h after the injection. Tissues from the heart, lung, liver, spleen, and kidney were immediately dissected, and then a substantial amount of blood was collected. Each tissue was wrapped in foil, properly weighed, and counted for 99mTc. Data points were corrected for physical decay of radioactivity. The distribution of the tissue was presented in percent injected dose per gram of wet tissue (%ID/g), which could be calculated by the percent injected (tissue activity/total activity dose) per gram of the wet tissue.

### Dosage Effect on Toxicity of PEG-CoFe_2_O_4_ in Mice

In this experiment, 21 mice were divided into seven groups (three mice/group). PEG-CoFe_2_O_4_ nanoparticles were injected in mice intravenously at different doses of 125, 250, and 350 μg/mouse (0.2 ml) with the control group that was treated with normal saline of 0.9%. In the treatment group, different doses of 125, 250, and 350 μg/mouse of curcumin were also injected intravenously in mice. The damage groups were killed after 24 h while the treatment groups were killed after 3 days. Blood was collected from the mice and centrifuged for about 10 min to obtain the serum. The serum contents of total bilirubin (TB), alanine aminotransferase (ALT), aspartate transaminase (AST), blood urea nitrogen (BUN), creatinine (CREA), and cystatin C (Cys-C) were measured. At the same time, the liver, the lung, the spleen, the kidney, and the heart were harvested immediately. These tissues were fixed in 10% buffered formalin and processed for the routine histology with hematoxylin and eosin. Microscopic observation of tissues was performed using an Olympus Microphot-CX41 microscope coupled with a digital camera.

## Results and Discussion

### TEM and XRD Analyses

**Fig. 1:**
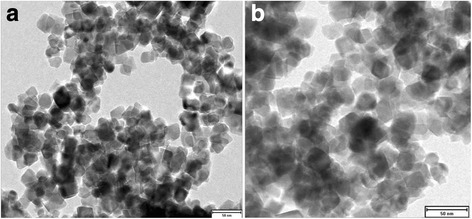
**a**, **b** Transmission electron microscopy (TEM) images of PEG-coated cobalt ferrite nanoparticles collected at different resolutions

**Fig. 2:**
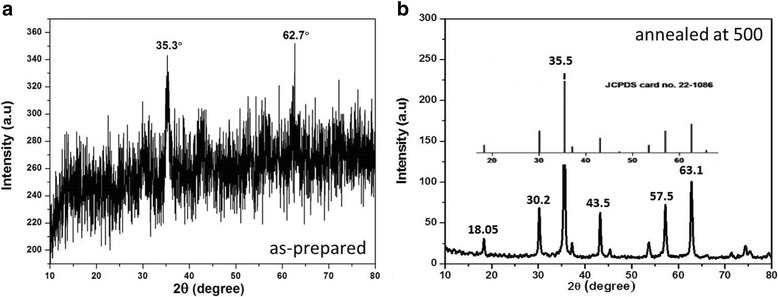
XRD results of the samples **a** as-prepared and **b** annealed at 500 °C. The inset shows the JCPDF card for cobalt ferrite. No extra peaks can be seen in the obtained XRD data

Morphological characterization was conducted using a JEOL JEM-1400 transmission electron microscope and X-ray diffractometer (Shimadzu XRD-7000) with copper K_α_ as the radiation source. Figure 1 displays the TEM images of PEG-coated cobalt ferrite nanoparticles with different resolutions (Fig. 1a, b), which confirms the successful formation of pure-phase PEG-coated cobalt ferrite nanoparticles with a particle size of about 24 nm. Figure 2 shows the X-ray diffraction analysis of the prepared nanoparticles. Figure 2a indicates the XRD results of the as-prepared samples, which show that the nanoparticles are mostly in amorphous form. However, when the samples were annealed at high temperature (i.e., 500 °C) for 6 h, it was found that the nanoparticles are turned into crystalline form, which can be seen in the XRD image presented in Fig. 2b. The mean crystallite size was calculated from the line broadening of the strongest peak in XRD analysis (Fig. 2b) using the Debye-Scherrer equation (*D* = *Kλ* / *β*cos*θ*) [13], which comes out to be ~ 22 nm. The positions and relative intensities of all the observed peaks in XRD pattern indicate that the crystalline structure favors the formation of cubic spinel structure of the nanoparticles according to the JCPDF card (card no. 20-1086) shown in the inset of Fig. 2b. All the peaks are indexed properly, and no extra peaks are seen in the XRD pattern, indicating that there are no impurities present in the samples. Both TEM and XRD results confirm the successful formation of crystalline nanoparticles of about 22–25 nm.

### Fourier Transform Infrared Spectroscopy, Raman, and TG Analyses

**Fig. 3:**
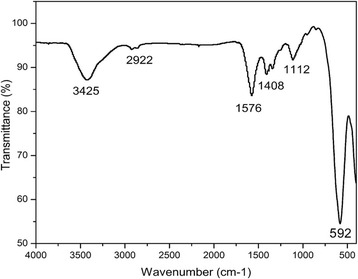
Fourier transform infrared spectroscopy (FTIR) employed in the range 500–4000 cm^−1^ to investigate the structural properties of the samples. The data confirms the PEG-coated cobalt ferrite nanoparticles

Fourier transform infrared spectroscopy (FTIR) was employed to investigate the structural properties and cation distribution of cobalt ferrite nanoparticles. Figure 3 shows the infrared spectrum of the samples taken at room temperature. Generally, cobalt ferrite has two strong absorption bands, *ʋ*_1_ and *ʋ*_2_, that appear in the range 400–600 cm^−1^ [14–16], which are quite obvious in our case. Higher band (*ʋ*_1_) corresponds to the intrinsic stretching vibrations of metal (M–O) at the tetrahedral lattice sites whereas the lower band (*ʋ*_2_) represents stretching vibrations of the metal ions at octahedral sites [14–16]. These results reveal the successful formation of cubic structured cobalt ferrite nanoparticles. From FTIR data, the peak shown at ~ 3400 cm^−1^ clearly indicates the PEG peak, which confirms the successful attachment of PEG with cobalt ferrite nanoparticles.

**Fig. 4:**
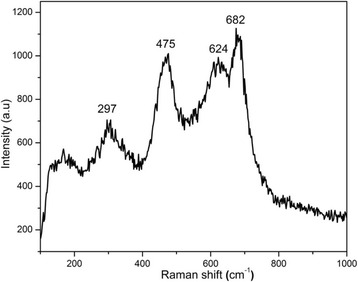
Room-temperature Raman spectrum of the samples collected in the 190–1000 cm^−1^ frequency range

Room-temperature Raman spectrum of the samples is shown in Fig. 4, which represents different peaks in the range 190–684 cm^−1^. The main peak at high frequency (684 cm^−1^) is the characteristic peak of the spinel ferrite attributed to A_1g_ mode which corresponds to the symmetric stretching of oxygen ions along the Fe–O bonds at tetrahedral sites [17]. The lower-frequency peaks also belong to spinel structured cobalt ferrite. The appearance of all these peaks in the Raman spectrum at appropriate energies confirms the successful formation of PEG-coated cubic CoFe_2_O_4_ nanoparticles.

**Fig. 5:**
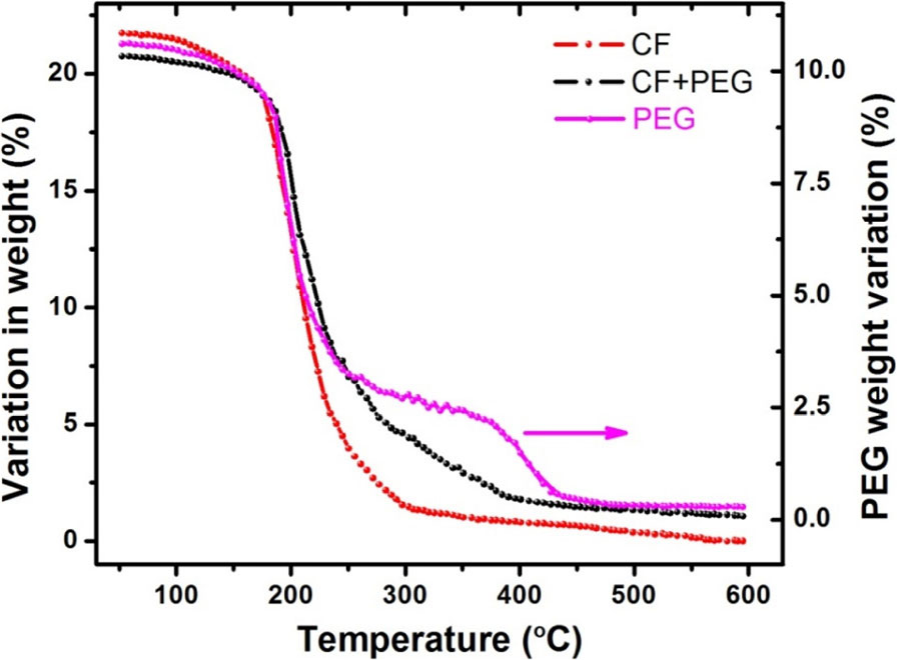
Thermogravimetric analysis (TGA) of pure CoFe_2_O_4_, PEG, and PEG-coated CoFe_2_O_4_ taken in the temperature range 50–600 °C

**Fig. 6:**
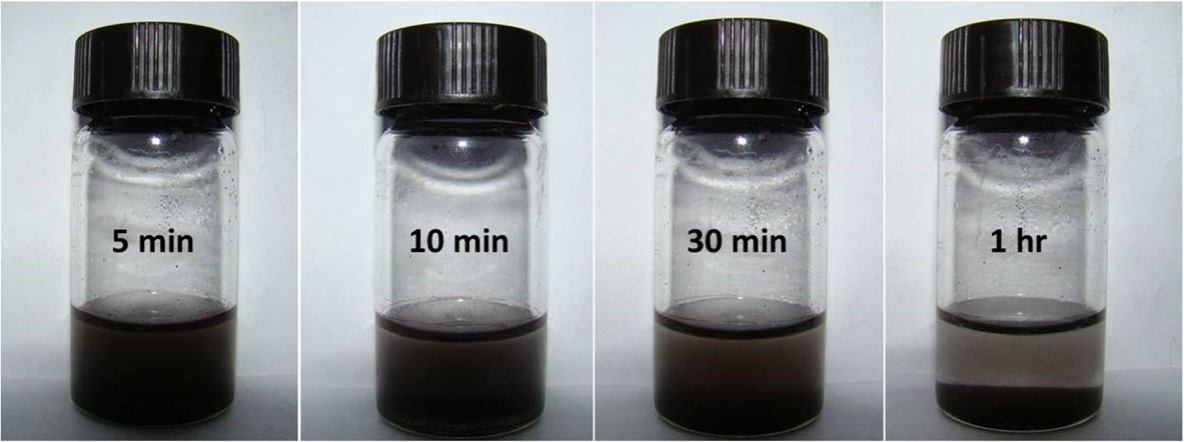
Solubility of PEG-CoFe_2_O_4_ nanoparticles in deionized water at different intervals (5, 10, 30, and 60 min) of time

Thermogravimetric analysis (TGA) of the samples (CoFe_2_O_4_, PEG, and PEG-CoFe_2_O_4_) was performed between 50 and 600 °C, and the results are shown in Fig. 5. These thermograms show that CoFe_2_O_4_ nanoparticles lose its weight in the range 200–300 °C, PEG loses its weight at a temperature below 400 °C, while PEG-CoFe_2_O_4_ loses its weight in the temperature range 200–400 °C. It is seen that the thermal stability of PEG is relatively poor (shown by the right side of the figure); however, the thermal stability of PEG-CoFe_2_O_4_ seems to be more than 80%. Pure cobalt ferrite nanoparticles are insoluble in water; however, it can be easily dissolved in water after coating with PEG due to its hydrophilic nature as shown in Fig. 6. It is seen in the figure that with passage of time, the particles are settled down at the bottom of the bottle, which is probably due to the gravity of nanoparticles. Figure 6 shows the time evolution of dissolution of PEG-coated cobalt ferrite nanoparticles. In our case, we fully dispersed the nanoparticles in saline before injecting into the body of the mice to ensure their proper delivery in different organs of mice.

### Biodistribution Studies

**Fig. 7:**
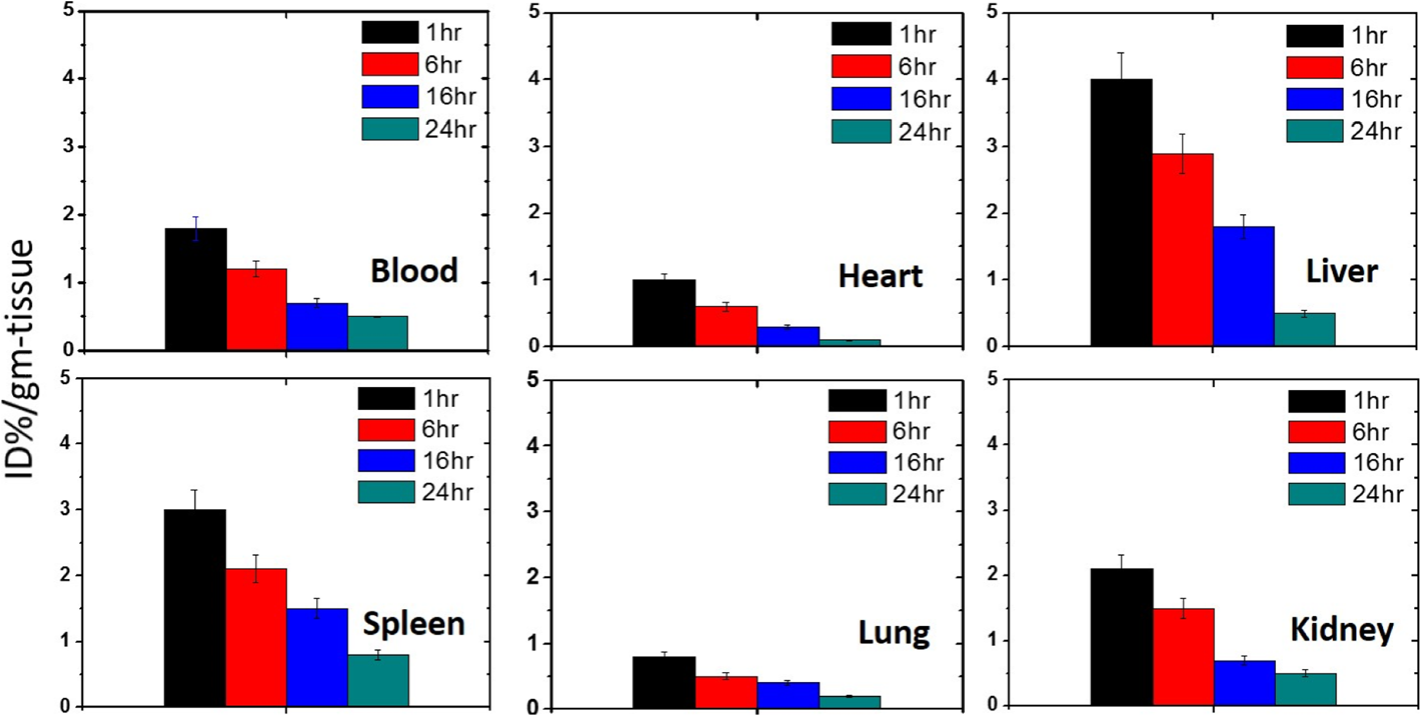
Biodistribution of PEG-CoFe_2_O_4_ nanoparticles in the blood, heart, liver, spleen, lungs, and kidney after different intervals (1, 6, 16, and 24 h) of exposure to mice. The error bars in the obtained data are shown in the figure

In order to accurately quantify the amount of the nanocarrier in each organ after administration in living organism, biodistribution of the 99mTc PEG-CoFe_2_O_4_ was performed in normal mice. It is seen that the uptake of PEG-CoFe_2_O_4_ is higher in the liver and spleen as shown in Fig. 7 and these results are the same with reference [18] where the uptake of radiolabeled cobalt ferrite at 1 h post injection is three times higher in the liver and spleen than that of other magnetic nanoparticles. The reason is that the tissues which bound to reticuloendothelial systems such as the liver and spleen largely take up these foreign particles, since these organs have Kupffer cells, which act as a cleaning function and play a major role in removing nano- and micromaterials from the body circulation by phagocytosis [19]. In this work, it is observed that the distribution of PEG-CoFe_2_O_4_ in tissues decreases with the passage of time, which means that PEG-CoFe_2_O_4_ nanoparticles are excreted with time through the urination process. The kidney is the excretory system for the nanoparticles through urine. In Fig. 7, the maximum biodistribution in the kidney is observed in 1 h [20]. Blood accumulation was high only immediately after the injection, indicating a relatively fast clearance of radioactivity from the body’s blood pool as shown in Fig. 7, which is similar to the case with iron oxide nanoparticles bearing PEG chains that have a prolonged presence in the blood pool [21, 22]. In addition, it was found that the biodistribution in the heart is very low, which is the same as reported in reference [23]. It is noticeable that the spleen is a primary site for old red blood cell destruction and subsequent recycling of hemoglobin-bound FE [18, 24]. It has been observed that over the time, slower but more efficient processes in the spleen are active and they are more capable of eliminating the nanoparticles from circulation, resulting in the increased tissue radioactivity concentrations after 1 h post injection. Lung uptake of PEG-CoFe_2_O_4_ was insignificant throughout our study as shown in Fig. 7. Similar work has been reported in reference [23]. This indicates that no microaggregates can be irreversibly trapped in the capillaries of lungs [23, 25, 26].

### Dosage Effect of PEG-CoFe_2_O_4_ on Toxicity

**Fig. 8:**
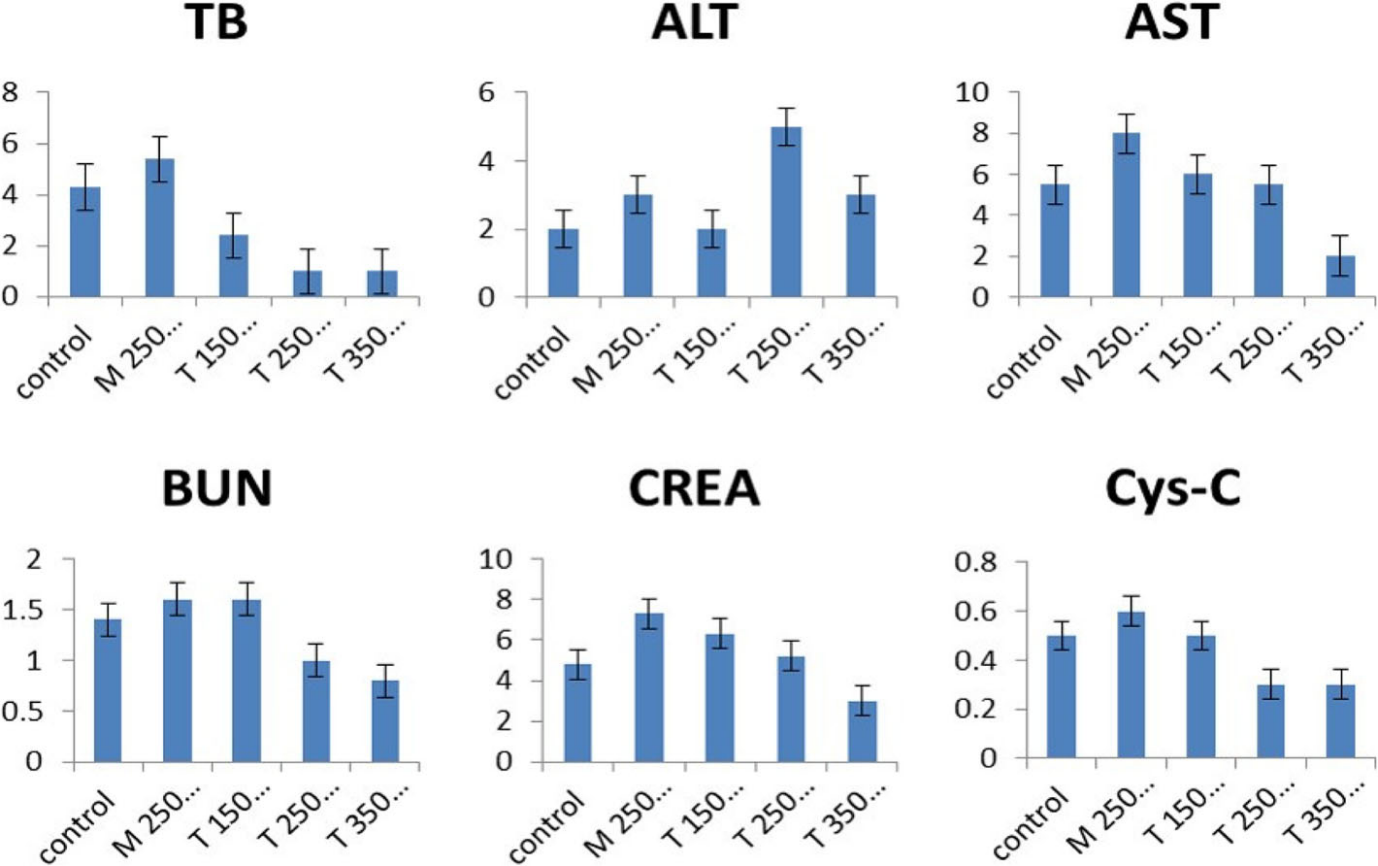
Biochemical indexes content in serum after different dosages (in μg) of PEG-CoFe_2_O_4_ exposure to mice with error bars shown in the figure

To reveal any potential toxic effects of PEG-CoFe_2_O_4_, we performed the biochemistry test on mice in vivo. For this purpose, we injected a mixed solution of saline and PEG-CoFe_2_O_4_ of different quantities (150, 250, and 350 μg) and sacrificed the mice after 24 h. For blood analysis, the blood was collected and centrifuged for about 10 min to obtain the serum. Various parameters were tested with focusing on liver and kidney function markers including Cys-C, CREA, ALT, AST, TB, and BUN. These parameters were then compared with control groups using SPSS software (*p* < 0.05 denotes a significant difference), and the results are shown in Fig. 8. A significant difference can be seen in ALT, BUN, and CREA-A between the exposure and control groups. It is seen that TB and Cys-C, which are mainly responsible for a biomarker of kidney function contents, were found to decrease significantly for the exposure of 150 μg per mouse of PEG-CoFe_2_O_4_ and it was found to increase for 250 μg per mouse dosage while it comes to the normal level for 350 μg per mouse. This suggests that up to a certain extent, the kidney function is affected by the exposure of PEG-CoFe_2_O_4_ but did not damage the tissues significantly. AST, which is a biomarker for the liver health, decreased significantly by the exposure to all dosages, which indicates that it can affect the liver function more as compared to the control group mice. From all these results, it is clear that a PEG-CoFe_2_O_4_ dosage of 250 μg/mouse exhibits relatively more damage. Therefore, for further analyses and tests in our experiment, we used 250 μg/mouse of PEG-CoFe_2_O_4_ dosage.

### Effect of Curcumin on Toxicity of PEG-CoFe_2_O_4_

**Fig. 9:**
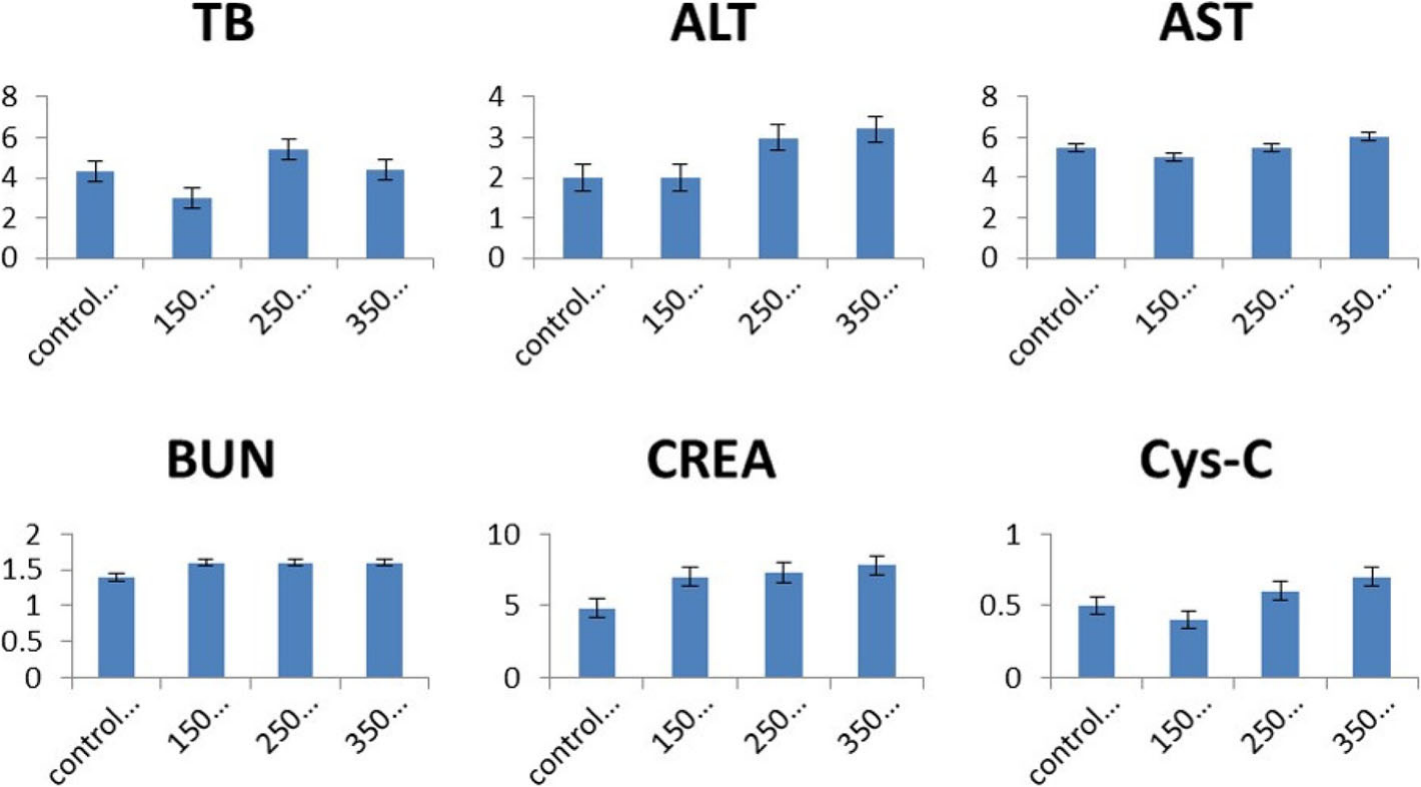
Biochemical indexes content in serum in the curcumin treatment group mice with error bars indicated in the graph

In this study, curcumin was employed to reduce the inflammation to the damage effect of PEG-CoFe_2_O_4_. In order to investigate the effect of curcumin on toxicity of PEG-CoFe_2_O_4_, biochemical indexes and tissue histology of mice were measured. These biochemical indexes include BUN, CREA, Cys-C, ALT, AST, and TB in the serum of the treatment group mice. It is seen that BUN, CREA, Cys-C, and AST show a significant decrease in different dosages of curcumin as compared to the exposure group, whereas in a dosage of 150 μg/mouse of curcumin, ALT, AST, and CREA come to the normal level as compared to the control group as shown in Fig. 9. In TB and ALT contents, all the dosages of curcumin show a significant decrease as compared to the exposure group of PEG-CoFe_2_O_4_. In Fig. 9, the results indicate that curcumin exhibits a positive treatment effect on the damage of PEG-CoFe_2_O_4_ in mice and different dosages of curcumin show better treatment effect. This work investigates the protective effect of curcumin against the serum level of liver enzymes (ALT and AST) and kidney enzymes (BUN, CREA, Cys-C, and TB). In this study, PEG-CoFe_2_O_4_ significantly increased the serum level of ALT, AST, BUN, CREA, Cys-C and TB enzymes as compared to the control group, which mostly approached the normal level after curcumin administration. Necrosis or cell membrane damage can cause the release of these enzymes into the blood. However, the serum level of these enzymes is associated with liver and kidney performance. In the groups that received curcumin, the amount of these enzymes was reduced, which indicates the protective effects of curcumin against the toxicity of PEG-CoFe_2_O_4_ nanoparticles. This is due to the antioxidant effect of curcumin that reduces the oxidative stress. Moreover, the TNF-α and IL-1 play a role in the induction of hepatic necrosis. Thus, curcumin can reduce the effect of toxicity by inhibiting the secretion of TNF-α and IL-1 by macrophages [11]. These findings are in agreement with other results reported in reference [27].

**Fig. 10:**
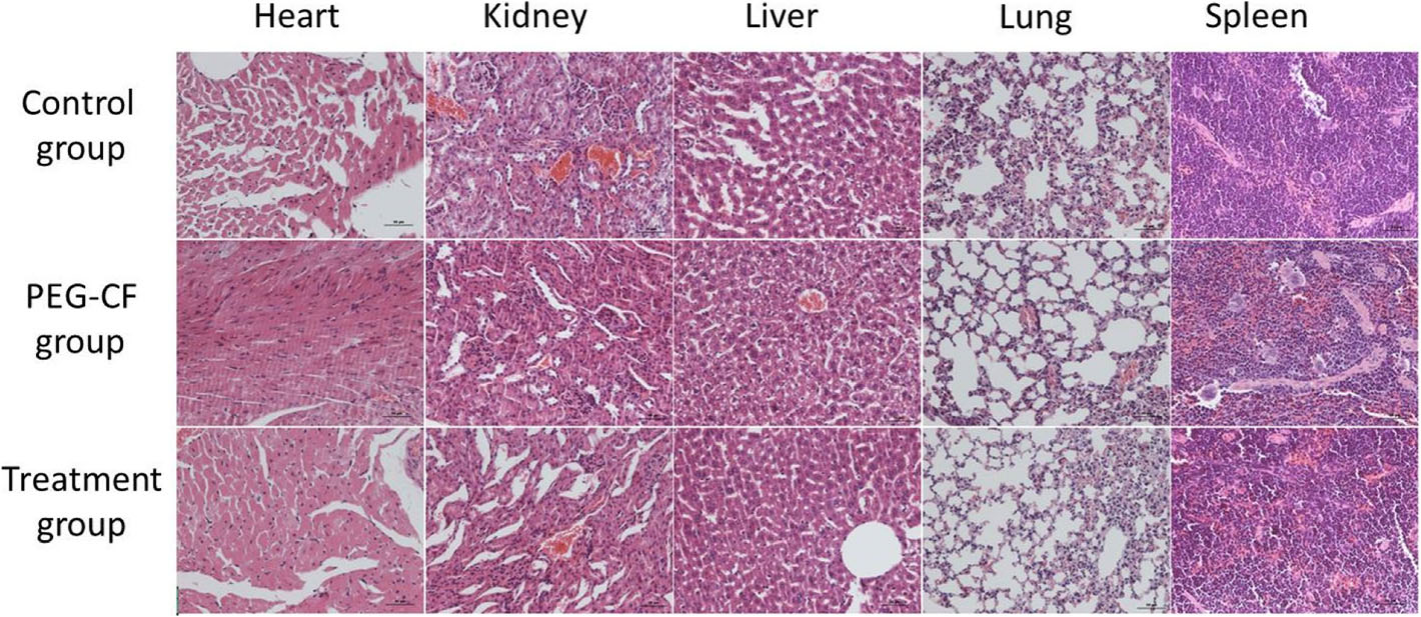
Histology sections of tissues after exposure of PEG-CoFe_2_O_4_ or curcumin to mice

The histopathological analysis of the liver, kidneys, and spleen was also performed in order to verify the possible toxic effects induced by the nanoparticle administration. The organs of each mouse were removed, treated in 10% formalin, and embedded in paraffin. Five-micrometer sections were stained with hematoxylin–eosin (H&E) and examined microscopically. The results show that no relevant histopathological changes were registered in analyzed organs that are shown in Fig. 10. The liver and spleen examinations showed that the organ architecture was not affected by the cobalt ferrite nanoparticle administration. This is due to the two possible reasons: firstly, the size of nanoparticles is relatively larger (i.e., 24 nm), and secondly, we gave a small dose of cobalt ferrite nanoparticles (i.e., 150, 250, and 350 μm) and killed the mice after 24 h. So, this only affects the function of the organs but did not possibly affect its architecture. This is similar to the case reported by authors in reference [28], where they gave 20 mg/kg (higher than our case) for 7 days. Similarly, in another case reported in reference [29], there were no histopathological changes monitored in the organs.

## Conclusion

In this work, we successfully fabricated 24-nm PEG-coated cobalt ferrite nanoparticles using a hydrothermal technique. Toxicity induced in various organs of mice using different dosages of PEG cobalt ferrite nanoparticles was explored in detail, and then its healing effect was studied using curcumin. Biological assays were performed to check the toxicity of CoFe_2_O_4_ nanoparticles. Positive changes were monitored in biochemical indexes after treatment with curcumin which either came to the normal level or decreased substantially. This study indicates that PEG-coated CoFe_2_O_4_ synthesized via a hydrothermal technique is a good model for a drug carrier and, curcumin, which is a natural chemical and possesses no side effects, could be utilized for the treatment of toxicity as well as for other diseases in living organisms.

## Abbreviations

ALT: Alanine aminotransferase
AST: Aspartate transferase
BUN: Blood urea nitrogen
CREA: Creatinine
Cys-C: Cystatin C
FTIR: Fourier transform infrared spectroscopy
H&E: Hematoxylin–eosin
NPs: Nanoparticles
Nrf2: Nuclear factor erythroid 2-related factor 2
PEG: Polyethylene glycol
TB: Total bilirubin
TEM: Transmission electron microscopy
TGA: Thermogravimetric analysis
TNF: Tumor necrosis factor
XRD: X-ray diffraction
